# Environmental Noise Pollution in the United States: Developing an Effective Public Health Response

**DOI:** 10.1289/ehp.1307272

**Published:** 2013-12-05

**Authors:** Monica S. Hammer, Tracy K. Swinburn, Richard L. Neitzel

**Affiliations:** 1 The Network for Public Health Law—Mid-States Region, The University of Michigan School of Public Health, Ann Arbor, Michigan, USA; 2 The Risk Science Center, The University of Michigan, Ann Arbor, Michigan, USA; 3 The Department of Environmental Health Sciences, The University of Michigan, Ann Arbor, Michigan, USA

## Abstract

Background: Tens of millions of Americans suffer from a range of adverse health outcomes due to noise exposure, including heart disease and hearing loss. Reducing environmental noise pollution is achievable and consistent with national prevention goals, yet there is no national plan to reduce environmental noise pollution.

Objectives: We aimed to describe some of the most serious health effects associated with noise, summarize exposures from several highly prevalent noise sources based on published estimates as well as extrapolations made using these estimates, and lay out proven mechanisms and strategies to reduce noise by incorporating scientific insight and technological innovations into existing public health infrastructure.

Discussion: We estimated that 104 million individuals had annual L_EQ(24)_ levels > 70 dBA (equivalent to a continuous average exposure level of >70 dBA over 24 hr) in 2013 and were at risk of noise-induced hearing loss. Tens of millions more may be at risk of heart disease, and other noise-related health effects. Direct regulation, altering the informational environment, and altering the built environment are the least costly, most logistically feasible, and most effective noise reduction interventions.

Conclusion: Significant public health benefit can be achieved by integrating interventions that reduce environmental noise levels and exposures into the federal public health agenda.

Citation: Hammer MS, Swinburn TK, Neitzel RL. 2014. Environmental noise pollution in the United States: developing an effective public health response. Environ Health Perspect 122:115–119; http://dx.doi.org/10.1289/ehp.1307272

## Introduction

Noise, or unwanted sound, is one of the most common environmental exposures in the United States (García 2001). In 1981, the U.S. Environmental Protection Agency (EPA) estimated that nearly 100 million people in the United States (about 50% of the population) had annual exposures to traffic noise that were high enough to be harmful to health (Simpson and Bruce 1981). However, despite the widespread prevalence of exposure, noise has historically been treated differently than pollutants of a chemical or radiological nature, and especially air pollution. Congress has not seriously discussed environmental noise in > 30 years, although noise exposure is a large public concern. For example, in New York City noise is consistently the number one quality of life issue, and authorities there received > 40,000 noise complaints in 2012 (Metcalfe 2013). Very few communities appear to consider the health risks of noise in their policy making (Network for Public Health Law 2013) despite the fact that the health effects of noise have been explored over many decades, and the body of evidence linking noise to various health effects is, therefore, more extensive than for most other environmental hazards (Goines and Hagler 2007; Passchier-Vermeer and Passchier 2000).

Even when cities and counties do address noise in their planning efforts, the results are disappointing. The Health Impacts Project (HIP) provides guidance for policy makers to identify the health consequences of potential projects by making public a national sample of health impact assessments (HIP 2013). Dozens of recent health impact statements in the HIP database have incorporated noise, but none appeared to assess changes in sleep disturbance, learning, hypertension, or heart disease. Although HIP does not provide a complete picture of U.S. health impact assessments, it does indicate that decision makers lack the information they need to protect communities from noise-related health effects. Environmental impact statements that calculate changes in noise levels also do not necessarily provide information about adverse health impacts resulting from these changes (U.S. Department of Transportation, Federal Highway Administration/Michigan Department of Transportation 2008).

In this commentary, we examine scientific and policy aspects of noise exposure. We first provide an overview of the relationship between high-impact health effects and noise. We then describe the most prevalent sources of noise and estimate prevalence of exposure. Finally, we explore policy approaches that can reduce the harmful effects of noise.

## Chronic Noise: A Biopsychosocial Model of Disease

**Figure 1:**
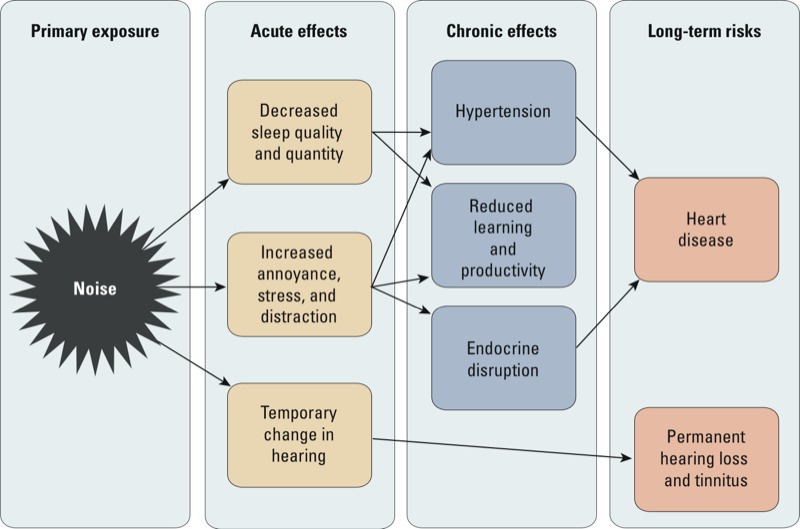
Select effects of noise.

Chronic environmental noise causes a wide variety of adverse health effects, including sleep disturbance, annoyance, noise-induced hearing loss (NIHL), cardiovascular disease, endocrine effects, and increased incidence of diabetes (Passchier-Vermeer and Passchier 2000; Sørensen et al. 2013). This commentary is not intended to provide a comprehensive review of all noise-related health effects, which is available elsewhere (Goines and Hagler 2007). Rather, we focus on several highly prevalent health effects: sleep disruption and heart disease, stress, annoyance, and NIHL (Figure 1). It is important to note that the levels of noise exposures associated with these health effects range widely; as a result, the prevention of different health effects involves specification of different exposure limits and metrics.

*Sleep and heart disease*. People in noisy environments experience a subjective habituation to noise, but their cardiovascular system does not habituate (Muzet 2002) and still experiences activations of the sympathetic nervous system and changes from deep sleep to a lighter stage of sleep in response to noise. The body’s initial startle response to noise is activation of the sympathetic (fight or flight) part of the nervous system, similar to the preparations the body makes just before waking in the morning. Although blood pressure normally drops during sleep, people experiencing sleep fragmentation from noise have difficulty achieving a nadir for any length of time because blood pressure rises with noise transients and heart rate increases with noise level (Haralabidis et al. 2008). Decreased quality and quantity of sleep elevates cardiovascular strain, which manifests as increased blood pressure and disruptions in cardiovascular circadian rhythms (Sforza et al. 2004).

Disordered sleep is associated with increased levels of stress hormones (Joo et al. 2012). Microarousals appear to be associated with increased lipids and cortisol levels, and feed into the same pathway of disordered sleep, even priming the neuroendocrine stress response in some individuals to be more at risk for disorders such as depression (Meerlo et al. 2008). Increased blood lipid, heart rate, blood pressure, and stress levels from noise lead to atherosclerosis, which is causally related to heart disease (Hoffman et al. 2013).

*Stress*. The effects of noise on conscious subjects are insidious and result at least in part from increased psychosocial stress and annoyance. Annoyance from continuous sound appears to vary substantially by individual (Babisch et al. 2013; Stansfeld 1992), and there are a number of factors that may influence annoyance (Babisch et al. 2012) and subsequent stress. Annoyance increases sympathetic tone, especially in noise-sensitive individuals (Sandrock et al. 2009), and may be the non–sleep-mediated pathway that is present in individuals with high occupational noise exposures who subsequently develop heart disease (Ha et al. 2011).

Environmental noise is not only a health risk to people who report being annoyed by noise, but these individuals are also at risk for additional health effects (Sandrock et al. 2009). Children in noisy environments have poor school performance, which leads to stress and misbehavior (Lercher et al. 2002). They also have decreased learning, lower reading comprehension, and concentration deficits (Stansfeld et al. 2005).

*NIHL*. Long-term exposures to noise levels > 75 dBA (U.S. EPA 1974) can cause metabolic changes in sensory hair cells within the cochlea, eventually leading to their demise (Heinrich et al. 2006) and increasing inability to perceive sound (e.g., NIHL). Neuronal destruction may also occur; in such cases, the ability to perceive sound may remain undiminished, but the ability to understand the meaning of sound deteriorates (Lin 2012). Extreme exposures can cause direct mechanical damage (acoustic trauma) to cochlear hair cells (Newby and Popelka 1992). Noise exposure is also associated with tinnitus (ringing in the ears) and hyperacusis. NIHL has traditionally been associated with occupational noise, but there is increasing evidence that music may play an important role as well (Lewis et al. 2013).

It is difficult to overstate the social cost of NIHL and its impact on quality of life. The additional effort required to process sound leads to fatigue, headaches, nervousness, depression, and anger (Hetu et al. 1993). Functional limitations associated with a compromised ability to communicate restrict mobility, self-direction, self-care, work tolerance, and work skills and increase isolation. Assistive technologies can aid some individuals, but in no way represent a cure.

Children with NIHL suffer from decreased educational achievement and impaired social–emotional development, score significantly lower on basic skills, and exhibit behavioral problems and lower self-esteem (Bess et al. 1998).

## Exposure Limits and Sources of Noise

*Exposure metrics and limits*. Because of the array of health effects caused by noise, and the relative importance of exposure timing for some health effects, a variety of exposure metrics and limits are in use today. The U.S. EPA recommends an average 24-hr exposure limit of 55 A-weighted decibels (dBA) to protect the public from all adverse effects on health and welfare in residential areas (U.S. EPA 1974). This limit is a day–night 24-hr average noise level (L_DN_), with a 10-dBA penalty applied to nighttime levels between 2200 and 0700 hours to account for sleep disruption and no penalty applied to daytime levels.

The U.S. EPA recommends a second exposure limit of 70 dBA to prevent hearing loss (U.S. EPA 1974). The limit is an equivalent continuous average exposure level over 24 hr [L_EQ(24)_]. Unlike the 55-dBA L_DN_ limit designed to protect against all long-term health effects, the 70-dBA limit considers daytime and nighttime exposures to be equally hazardous to hearing. This 24-hr limit is equivalent to a 75-dBA 8-hr workday exposure, with no noise exposure (i.e., noise < 70 dBA) during the remaining 16 hr.

The U.S. EPA recommendations—adopted in 1974 and mirrored by the World Health Organization (WHO) (Berglund et al. 1999)—may be considered a truly “safe” level for protection against hearing loss. In contrast, the U.S. Occupational Safety and Health Administration’s 8-hr workplace regulation of 90 dBA may result in a 25% excess risk of hearing impairment among workers exposed over a working lifetime [National Institute of Occupational Safety and Health (NIOSH) 1998].

Other limits may be needed or appropriate for preventing additional health effects not described here or for emerging sources of noise (e.g., wind turbines) that are substantially different from historical noise sources. For example, the WHO recently adopted a set of health-based guidelines for nighttime noise exposure that are much lower than previously recommended levels (WHO 2009).

*Sources of noise*. Primary sources of noise in the United States include road and rail traffic, air transportation, and occupational and industrial activities [National Academy of Engineering (NAE) 2010]. Additional individual-level exposures include amplified music, recreational activities (including concerts and sporting events), and firearms. Personal music player use appears to be common among adolescents (Kim et al. 2009; Vogel et al. 2011) and may involve potentially harmful sound levels (Breinbauer et al. 2012). Exposures from recreational activities and music are not “noise” in the sense of being unwanted sound, but adverse health effects are possible even from desirable sounds.

## Prevalence of Harmful Noise Exposure

Data on the prevalence of noise exposures in the United States are dated and inadequate. The most recent national surveys of community and occupational noise exposures occurred in the early 1980s (NIOSH 1988; Simpson and Bruce 1981). Current estimates of workers exposed to “hazardous” levels of workplace noise (an 8-hr L_EQ_ of ≥ 85 dBA) range from 22 to 30 million (NIOSH 2001; Tak et al. 2009). This wide range in estimates for the working population, which is more closely tracked than the general public, should give some indication as to the tremendous uncertainty in community estimates.

The limited data available suggest that a substantial portion of the U.S. population may be at risk of noise-related health effects and that modern 24-hr societies are increasingly encroaching on “quiet” periods (e.g., night). An annual level of 55- to 60-dBA L_DN_ may increase risk of hypertension (van Kempen and Babisch 2012). In 1981, Simpson and Bruce (1981) estimated that at least 92.4 million people (46.2% of the U.S. population) were exposed at or above this level. Applying the 1981 U.S. EPA estimate of exposure prevalence to the current U.S. population (315 million in March 2013) (U.S. Census Bureau 2010), and assuming noise levels have not changed since then, we estimate that at least 145.5 million people were at potential risk of hypertension due to noise in 2013. Lower levels (e.g., 50–55 dBA, to which a larger fraction of the population is exposed) may increase risk of myocardial infarction (Willich et al. 2006).

Recent studies of individuals’ noise exposures (Flamme et al. 2012) indicate that a substantial fraction of U.S. adults may be exposed to noise levels above the U.S. EPA 70-dBA L_EQ(24)_ limit. Neitzel et al. (2012) sampled > 4,500 adults in New York City and estimated that 9 of 10 exceeded the recommended U.S. EPA limit. The Neitzel et al. (2012) study is the most comprehensive quantitative estimate of annual noise exposures in a large sample of U.S. residents in decades, and it represents a basis for developing contemporary estimates of urban U.S. noise exposures.

There are 16 metropolitan statistical areas in the United States with a population of > 4 million for which the New York City estimates might be considered representative. These areas comprised a total population of 80,621,123 in 2012 (U.S. Census Bureau 2010), or 25.6% of the U.S. population. By applying the New York City exposure prevalence estimates of Neitzel et al. (2012) to these 16 largest urban agglomerations, we estimate that at least 72.6 million urban U.S. residents were exposed to annual L_EQ(24)_ levels of > 70 dBA in 2010. By comparison, the U.S. EPA estimated in 1981 that 66 million people, or 33% of the U.S. population (not just urban dwellers), were exposed above the recommended limit (Simpson and Bruce 1981). Applying the 1981 U.S. EPA estimate to 2013 census data, and again assuming no change in noise levels over that time, we estimate that 104 million individuals had annual L_EQ(24)_ levels of > 70 dBA in 2013 and were at risk of NIHL and possibly other noise-related health effects. Unfortunately, given the lack of assessment of noise exposure in health surveillance programs in the United States, it is difficult to evaluate these estimated health impacts against observed health effects, and for some health effects metrics other than the L_EQ(24)_ (e.g., the L_DN_) are likely more appropriate.

## Health Protection Policy

Given the substantial exposures to noise in the United States, the severity of associated health consequences, and the limited power of the public to protect themselves, there is a clear need for policy aimed at reducing noise exposures. Because noise is expected to rise with increasing urbanization (García 2001), policy leaders need to explore the use of law as a practical tool to manage and reduce noise exposures. Here we highlight the interventions we believe hold the most promise for policy leaders. We first explain how noise can be integrated into the federal public health agenda and then explore the ways state and local governments may use the law to respond to and reduce noise.

*The federal public health agenda*. The United States National Prevention Strategy (NPS) can provide leadership by putting noise on the national health policy agenda. The NPS brings together 17 federal agencies (including the Departments of Transportation, Health and Human Services, Education, and Labor as well as the U.S. EPA) to provide a foundation for the nation’s prevention goal delineated under the Affordable Care Act: to increase the number of Americans who are healthy at every stage of life through focus on wellness and prevention (National Prevention Council 2011). Two of NPS’s priorities are *a*) to promote healthy and safe community settings that prevent injury, and *b*) to empower people in ways that support positive physical and mental health. In addition, some of the objectives of the Department of Health and Human Services (DHHS), as articulated in their Healthy People 2020 goals, are to decrease the proportion of adolescents who have NIHL, reduce new cases of work-related noise-induced hearing loss (DHHS 2013a), increase cardiovascular health, and reduce coronary heart disease deaths (DHHS 2013b). These federal objectives, designed to encourage collaboration and improve decision making, can also be used to coordinate and measure the impact of prevention strategies set forth below. Although there is a large range of options for addressing noise exposures in the United States (NAE 2010), we believe that direct regulation and altering the informational environment are the least costly, most logistically feasible, and most effective federal-level noise reduction interventions.

Source control through direct regulation. Direct regulation that sets maximum emission level for noise sources is the only intervention that guarantees population-level exposure reductions. The NPS supports proven strategies, and source reduction is the most cost-effective intervention to protect health (García 2001). There is already evidence of the great potential for this approach in the United States: annual U.S. air transport noise exposures > 65 dBA L_DN_ have seen a remarkable 90% reduction since 1981 (from affecting 4% of the population in 1981 to 0.015% in 2007) despite a sixfold increase in number of person-miles travelled by air. This reduction can be attributed in large part to direct federal regulation, and subsequent technological improvements of jet engines (Waitz et al. 2007).

The regulatory scheme for direct source regulation is straightforward. Congress gave power to the U.S. EPA to regulate noise emitted from construction equipment, transportation equipment, any motor or engine, and electrical or electronic equipment in the Noise Control Act (NCA) of 1972 (NCA 1972a). Between 1972 and 1981 the U.S. EPA Office of Noise Abatement and Control (ONAC) led efforts which resulted in noise emission limits on air compressors, motorcycles, medium and heavy trucks, and truck-mounted waste compactors. An attempt to regulate lawn mowers was not well received (Shapiro 1991), and the agency lost funding in 1981, when the ONAC budget was $12.7 million ($32.5 million in 2013 dollars) (U.S. EPA 1982).

The U.S. EPA could resume noise control work with support from Congress and the NPS. The majority of the U.S. EPA’s funding ($7.1 billion in 2012) consists of discretionary appropriations from Congress, which means that the U.S. EPA can exercise the full scope of its regulatory authority under the NCA at any time. However, U.S. EPA funding in real dollars adjusted for inflation peaked in 1978 (Congressional Research Service 2012), so it is likely that the U.S. EPA will resume activity on noise control only when Congress and the NPS support their efforts.

Altering the informational environment. The NPS seeks to empower individual decision making by addressing barriers to the dissemination and use of reliable health information. Altering the informational environment enables informed choice in partnership with direct regulation. Without source control, changing the informational environment can only offer limited reductions in noise because individuals often lack control over significant noise sources. However, several interventions have the potential to drastically alter the informational environment.

## Product Disclosure

Labels that disclose the noise emitted from products promote informed consumer choice. Mandatory labeling of noise emissions is required for certain products in China, Argentina, Brazil, and the European Union (NAE 2010). Disclosure will inform consumer choice only if the consumer understands the implications of what the label discloses, so we discuss product disclosures with the assumption that they will be accompanied by education.

The NCA requires that the U.S. EPA adopt regulations that label products that emit noise capable of adversely affecting the public health or welfare (NCA 1972b). The U.S. EPA implemented this mandate only for portable air compressors, even though there are many other, more noisy products, including children’s toys (Hawks 1998). Individuals without access to education may still experience some benefit from product disclosures that are easily understood, such as warnings based on red, yellow, and green colors. The U.S. EPA could resume its work mandating disclosures with NPS leadership and Congressional funding.

## Mapping

Geographic noise maps alter the informational environment and are one way to ensure that noise control policy is based on objective and accurate information. The NPS seeks to expand and increase access to information technology and integrated data systems. Governments in the European Union have already prepared noise maps of roads, railways, and airports (Commission to the European Parliament and the Council 2011). Although the U.S. government does not map noise levels to protect the public, the National Oceanic and Atmospheric Administration (2012) has created a noise map of the world’s oceans to investigate the impact of noise on marine species. Cities such as San Francisco have mapped traffic noise, but most cities and states would need federal support and guidance to initiate comprehensive mapping. Measurement and mapping of noise levels—following the example of the CDC’s air and water quality databases—would identify priorities for additional evaluation and help inform protective measures. Congress can appropriate funding to the U.S. EPA, ONAC, or CDC to support this work. However, mapping efforts will require a substantially increased and ongoing noise monitoring effort.

*State and local action*. The NPS addresses the complex interactions between federal, state, tribal, local, and territorial policies addressing community environments. The NCA was first enacted at the behest of industry trade groups that argued that national standards would protect manufacturers from the imposition of disparate and inconsistent state and local standards. However, after it was enacted, industry groups asked for a defunding of the NCA by asserting that it was best to control noise at the local level (Shapiro 1991).

State and local governments can enact regulations on sources of noise not already regulated by the U.S. EPA or another federal agency. Theoretically, a mixed system where federal and state jurisdiction overlap increases functionality. In the case of noise control, however, few states and localities attempt direct regulations because they do not have sufficient market power and resources and because of preemption challenges from other law (Air Transport Association of America *v.* Crotti 1975). Municipal regulation evolved into noise ordinances that regulate the timing and intensity of noise, are expensive and difficult to enforce, and have not proven to be effective at reducing noise (Dunlap 2006).

Given these considerations, we believe that the most cost-effective legal interventions at the state and local levels are through *a*) spending and procurement, and *b*) altering the built environment.

Spending and procurement. A number of municipal noise sources, including emergency sirens, transit vehicles, garbage and street maintenance equipment, and construction equipment (Bronzaft and Van Ryzin 2007), may be reduced through careful purchasing and contractual agreements. Some countries go so far as to require contractors to pay for temporary relocation of citizens seeking relief from construction noise (BSM 2012). Adoption of procurement policies intended to reduce community noise is an opportunity for government to lead by example (Perdue et al. 2003).

Altering the built environment. The NPS recommends that governments take steps to ensure safe and healthy housing because health suffers when people live in poorly designed physical environments (Perdue et al. 2003). Although altering the built environment can influence individual noise exposures, it often does not reduce noise source levels. In addition, it can be construed as inherently inequitable because the recipients of noise bear the burden of exposure reduction, and those creating the noise continue to have no incentive to reduce emissions. Therefore, this intervention requires thorough analysis and careful planning.

Sustainable building design programs, such as Leadership in Energy and Environmental Design (LEED), offer the possibility of achieving noise reductions through good acoustical design (U.S. Green Building Council 2013). LEED standards incorporate American National Standards Institute recommendations regarding background noise and encourage sound-absorptive finishes to limit reverberation in schools (U.S. Green Building Council 2010). Improvements in construction materials, siting considerations (e.g., siting sensitive structures such as homes and schools well away from noise sources such as high traffic roads and hospitals), and design can have a dramatic impact on noise levels inside buildings—and improve the occupants’ quality of life in the process.

Although the Federal Highway Administration does not currently provide federal funding for low-noise pavement (NAE 2010), such pavement can reduce noise by up to 6 dB in areas where vehicles travel at speeds > 35 miles/hr. For slower traffic, planning can reduce high noise from delivery trucks within city limits by encouraging adoption of smaller electric delivery vehicles. This scheme has already been implemented in several other countries (Allen et al. 2012) and also has the potential to reduce air pollution and traffic fatalities.

## Conclusion

We have identified a number of opportunities to lower noise exposures and ultimately improve public health while additional research is being conducted. Updated national-level estimates of individual noise exposures are needed; our use of 1981 U.S. EPA data introduces a substantial amount of uncertainty into our estimates and highlights the need for an updated national survey of noise exposures in the United States. Although prevention of different health effects will require additional research to identify appropriate exposure limits, once informed and supported by ongoing research, federal leaders can focus on lowering noise at its source, and states can prioritize altering the built environment. Meanwhile, local government can adjust their procurement policies and encourage building approaches that reduce community noise.

## Correction

In the manuscript originally published online, the reported annual noise level that may increase risk for hypertension, the reported estimate of the number of people exposed at or above the annual noise level, and the authors’ estimate of the number of people at potential risk of hypertension due to noise in 2013 were incorrect in the second paragraph of the “Prevalence of Harmful Noise Exposure” section. They have been corrected here.
