# Correction: Environmental Noise Pollution in the United States: Developing an Effective Public Health Response

**DOI:** 10.1289/ehp.1307272-erratum

**Published:** 2013-12-05

In the manuscript originally published online, the reported annual noise level that may increase risk for hypertension, the reported estimate of the number of people exposed at or above the annual noise level, and the authors’ estimate of the number of people at potential risk of hypertension due to noise in 2013 were incorrect in the second paragraph of the “Prevalence of Harmful Noise Exposure” section. They have been corrected here.

